# Hemodynamics and pathology of an enlarging abdominal aortic aneurysm model in rabbits

**DOI:** 10.1371/journal.pone.0205366

**Published:** 2018-10-12

**Authors:** Hongmei Chen, Yonghua Bi, Siyeong Ju, Linxia Gu, Xiaoyan Zhu, Xinwei Han

**Affiliations:** 1 Department of Histology and Embryology, College of Basic Medicine, Zhengzhou University, Zhengzhou, China; 2 Department of Ultrasound, Zhengzhou Central Hospital Affiliated to Zhengzhou University, Zhengzhou, China; 3 Department of Interventional Radiology, the First Affiliated Hospital of Zhengzhou University, Zhengzhou, China; 4 Department of Mechanical and Materials Engineering, University of Nebraska-Lincoln, Lincoln, Nebraska, United States of America; Max Delbruck Centrum fur Molekulare Medizin Berlin Buch, GERMANY

## Abstract

Hemodynamics may play an essential role in the initiation and progression of abdominal aortic aneurysm (AAA). We aimed to study the mechanism of self-healing process by the changes of hemodynamics and pathology in an enlarging AAA in rabbits. Seventy-two rabbits were randomly divided into three groups. Rabbits underwent extrinsic coarctation and received a 10-minute elastase incubation in Group A and Group B. Absorbable suture used in Group A was terminated by balloon dilation at week 4. Diameter was measured after 1, 3, 5, and 15 weeks, computational fluid dynamics analysis was performed at week 3 and week 15. Rabbits were sacrificed after 1, 5, and 15 weeks for pathological and quantitative studies. The higher velocity magnitude, intensified bulk flow and obvious vortex formation were observed in Group A at week 3 instead of week 15. Both low wall shear stress and high relative residence time increased in Group B, however, high oscillatory shear index had relatively less increase compared with Group A. Aortic diameter reached a plateau at 5 weeks in Group A, which was significantly lower than in week 15 in Group B. Intimal hyperplasia, intima-media thickness increased significantly in Group A at week 5, significantly higher than in week 15 in Group B. Marked destruction of elastin fibers and smooth muscle cells occurred at week 1, and increased significantly at week 15 in Group A. Aneurysm exhibited strong expression of matrix metalloproteinase 9 and mouse anti-rabbit macrophage 11 at week 1, and showed a tendency to decrease. Matrix metalloproteinase 2 expression decreased significantly in Group B at week 15 compared with week 5 and Group A. In conclusion, the self-healing of rabbit AAA may attributed to the regeneration of smooth muscle cells. The turbulence flow caused by coarctation is associated with continuous growth of rabbit AAA and prevents the self-healing phenomenon.

## Introduction

Abdominal aortic aneurysm (AAA) is characterized by chronic remodeling of aortic wall tissue, however, its pathogenesis remains poorly understood. [1] Animal models have been developed to mimic human AAA disease, [2] which included genetically predisposed AAA model, chemical injury AAA model, and hemodynamically-induced AAA model. Although the chemical injury AAA model has been popular used in animal studies, [3–9] this model does not always induce stable AAA. [10–12] It has been reported that elastase-induced AAA in rabbit heals spontaneously. [8] This performance is quite different from human AAA. Thus, the enlarging AAA models have been successfully induced to overcome the self-healing phenomenon. [4, 13] The different outcomes of elastase-induced AAA and an enlarging AAA indicated that hemodynamic change may play an essential role in the initiation and progression AAA in rabbits. In the current study, we aimed to study the mechanism of self-healing process by the changes of hemodynamics and pathology in an enlarging AAA in rabbits.

## Materials and methods

### Experimental groups

Seventy-two New Zealand white rabbits were randomly divided into three groups. Rabbits underwent an extrinsic coarctation below the renal artery and received a 10-minute incubation of elastase with no pressure (Shanghai Kayon Biological Technology Co., Ltd, China) in Group A and Group B. A polyglycolic acid suture (Pudong gold ring medical supplies limited, Shanghai, China) was used for inducing coarctation, which will be terminated by balloon dilation at week 4 in Group A. Non-absorbable suture was used in Group B, and then be ballooned at the same pressure as the group A, but without correcting the coarctation. The sham operation was performed as a control in Group C, and the same dose of physiological saline was used.

### AAA model induction, angiography, and balloon dilation

**Fig 1:**
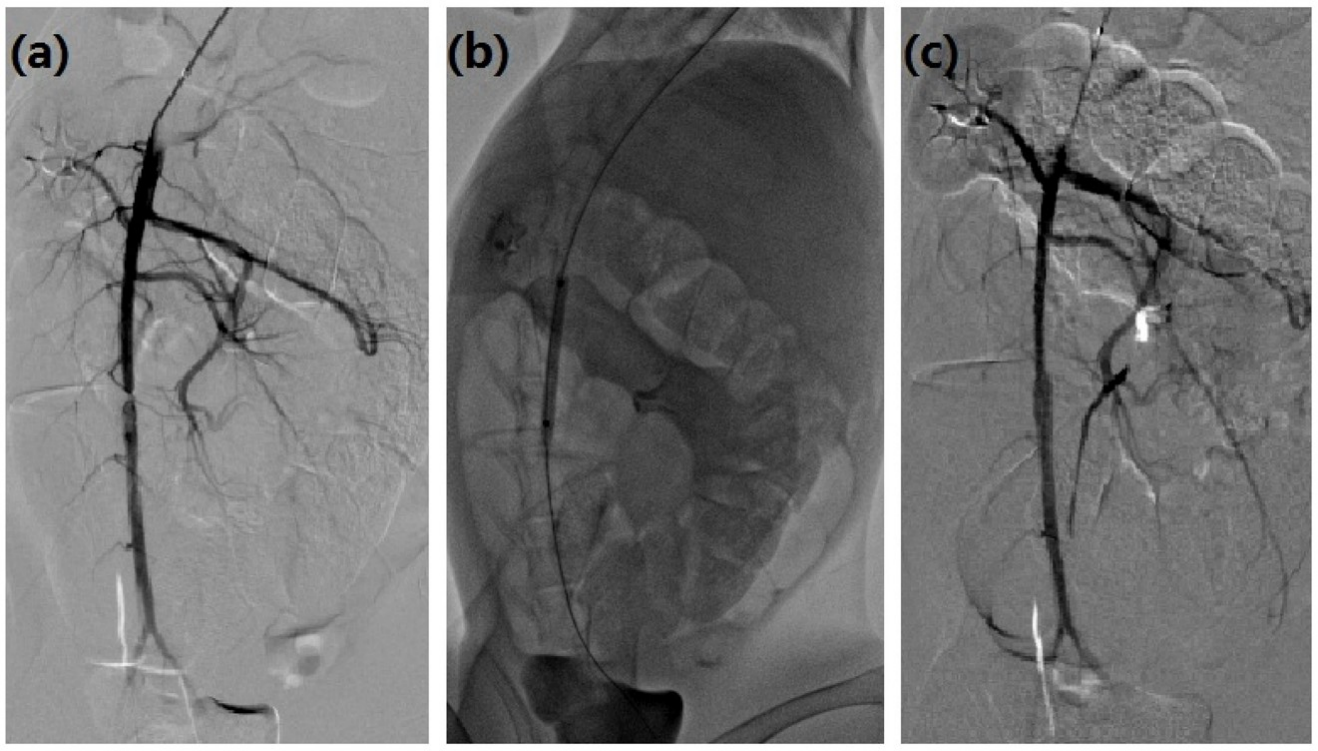
Balloon dilation procedure in Group A. An obvious coarctation was shown 4 weeks later in Group A (a); Balloon was dilated to terminate coarctation at week 4 (b); Coarctation was terminated after dilation (c).

This study was conducted under the approval of the Animal Experimental Ethics Committee of Zhengzhou University. All surgery was performed under sodium pentobarbital anesthesia and all efforts were made to minimize suffering. Aneurysm was induced according to previous report [4]. A 2-cm segment of infrarenal abdominal aorta was dissociated and incubated with porcine pancreatic elastase (60 μl, 1 unit/μl) for 10 minutes, and then a ligature was performed proximal segment in Group A and Group B. All rabbits underwent 5s Digital subtraction angiography (DSA) scanning (Siemens Artis Zee, Germany) at 1, 3, 5, and 15 weeks. [14] The diameter measurement and 3D-DSA analysis were performed with RadiAnti DICOM Viewer 3.4.2 (Medixant Company, Poland). Four weeks later, all rabbit in Group A underwent balloon dilation. The right common carotid artery was cannulated and a 4F sheath was indwelled. The 3.5 F micro catheter was introduced via a 0.014 in micro guide wire, and angiography was performed to show the coarctation. A 3 mm × 8 mm balloon catheter was introduced and dilated to terminate the coarctation (Fig 1).

### Histopathology analysis

Rabbits were sacrificed for histopathology after 5s DSA at 1, 5, and 15 weeks. Aneurysm tissues were fixed with 4% buffered paraformaldehyde and embedded in paraffin for histopathology analysis. The hematoxylin-eosin and elastic van-Gieson staining were performed to show the morphology and elastic lamellae distribution. Aortic lumen perimeters (media thickness, intima thickness and intima-media thickness) were measured by Image Pro-Plus 6.0 software (Media Cybernetics, Rockville, MD) under × 400 magnifications.

### Immunohistochemistry and immunofluorescence

SP method was used for immunohistochemistry according to the manufacture’s protocol. After incubation with 1% H_2_O_2_ in methanol and 10% goat serum for 30 min, the primary antibodies to MMP2 (ab2462, Abcam, Hong Kong, China), MMP9 (ab58803, Abcam, Hongkong, China), and monoclonal mouse anti-rabbit macrophage (Clone RAM11, Dako, Denmark) were incubated overnight at 4 °C. Sections were incubated with biotinylated anti-mouse second antibody for 20 min, and visualized with diaminobenzidine tetrahydrochloride. Immunofluorescence was used to detect the assembly of smooth muscle cells (SMCs). Sections were incubated overnight with mouse monoclonal anti-alpha-smooth muscle actin (A2547, Sigma-Aldrich (Shanghai) Trading Co., Ltd, China), and subsequently incubated with horseradish peroxidase-conjugated goat anti-mouse IgG. Semiquantitative analyses for SMCs content was calculated as the mean area of 6 fields per rabbit.

### Western blot for MMP2, MMP9 and RAM11

The RIPA protein lysis buffer (Code P0013B, Beyotime Biotechnology, Shanghai, China) was used to lyse aneurysm tissues. Total protein was separated by 10% SDS-PAGE and transferred to a polyvinylidene difluoride membrane (Millipore Corporation, MA, USA). After incubation with 5% milk solution, the membrane was probed with primary antibodies (MMP2, MMP9 and RAM11) overnight. The membrane was incubated with HRP-conjugated secondary antibody for 2 h, and the protein band was visualized by an enhanced chemiluminescence kit (Applygen Technologies Inc., Beijing, China). The β-actin served as an internal control. Densitometric analysis of protein bands was performed via public domain software NIH Image version 1.61.

### Computational fluid dynamics analysis

Computational fluid dynamics analysis of the abdominal aorta, including streamline, velocity and pressure distribution, wall shear stress (WSS), oscillatory shear index (OSI), and relative residence time (RRT), were performed at two time points during the progression of AAA. The relationship between the aforementioned hemodynamic parameters and the initiation and progression of AAA were investigated. Animal-specific computational fluid dynamics models were reconstructed from CT images using Mimics software (Materialise, Leuven, Belgium). Reconstructed AAA models from Group A and Group B were investigated. The method is described as below.

#### Method of CFD analysis

The model was meshed with approximately 2.38 million elements and 0.58 million nodes using the commercial software ANSYS ICEM (ANSYS Academic Research 17.2). Five layers of hexahedral elements were used to better capture the near wall phenomena, and tetrahedral meshes were used for the lumen of artery. A pulsatile velocity profile [15] was prescribed at the inlet. The outlet pressure was assumed as a constant of 100 mm Hg. No-slip boundary condition was implemented at the rigid wall. Blood was assumed as an incompressible Newtonian liquid with a density ρ of 1060kg/m^3^ and dynamic viscosity μ of 0.0035Pa•s.

The blood flow was governed by the mass and momentum conservation equations:

$$\text{Mass}:\nabla \cdot \vec{V}$$

$$\text{Momentum}:\rho \left[\frac{\partial \vec{V}}{\partial t}+\left(\nabla \cdot \vec{V}\right)\vec{V}\right]=\;-\nabla P\;+\;\mu \nabla^{2}\vec{V}$$

where ρ is the fluid density, V, P, and μ are velocity, pressure, and viscosity of a fluid respectively. The CFD models were solved using commercial package ANSYS FLUENT 17.2.

#### Hemodynamic parameters

The wall shear stress is the drag force per unit area casued by blood flow across the artery wall. Time-averaged WSS (TAWSS) over one cardiac cylcle was used in this work:

$$\text{TAWSS}=\frac{1}{T}\int_{0}^{T}\left|{\text{WSS}}_{\text{i}}\right|dt$$

where *WSS*_*i*_ is the instantaneous shear stress vector and T is the duration of the cycle. The OSI is a nondimensional parameter that measures the directional change of WSS during a cardiac cycle [3]

$$\text{OSI}=\frac{1}{2}\left(1-\frac{\left|\int_{0}^{T}{\text{WSS}}_{\text{i}}dt\right|}{\int_{0}^{T}\left|WSS_{i}\right|dt}\right)$$

The OSI was often used to describe the disturbance of a flow field. The RRT incoporates both WSS and OSI and represents the residence time of blood near the artery wall. [16]

$$\text{RRT}\sim {\left[(1-2\cdot \text{OSI})\cdot \text{TAWSS}\right]}^{-1}$$

### Statistical analysis

Data were expressed as means ± SD. One-way ANOVA and two-way ANOVA, followed by Bonferroni post hoc tests, were used for statistical analysis (Prism 5.0, GraphPad Software, Inc., SanDiego, CA., USA). Statistical significant differences were considered if *p* value was less than 0.05.

## Results

### Hemodynamic performance

**Fig 2:**
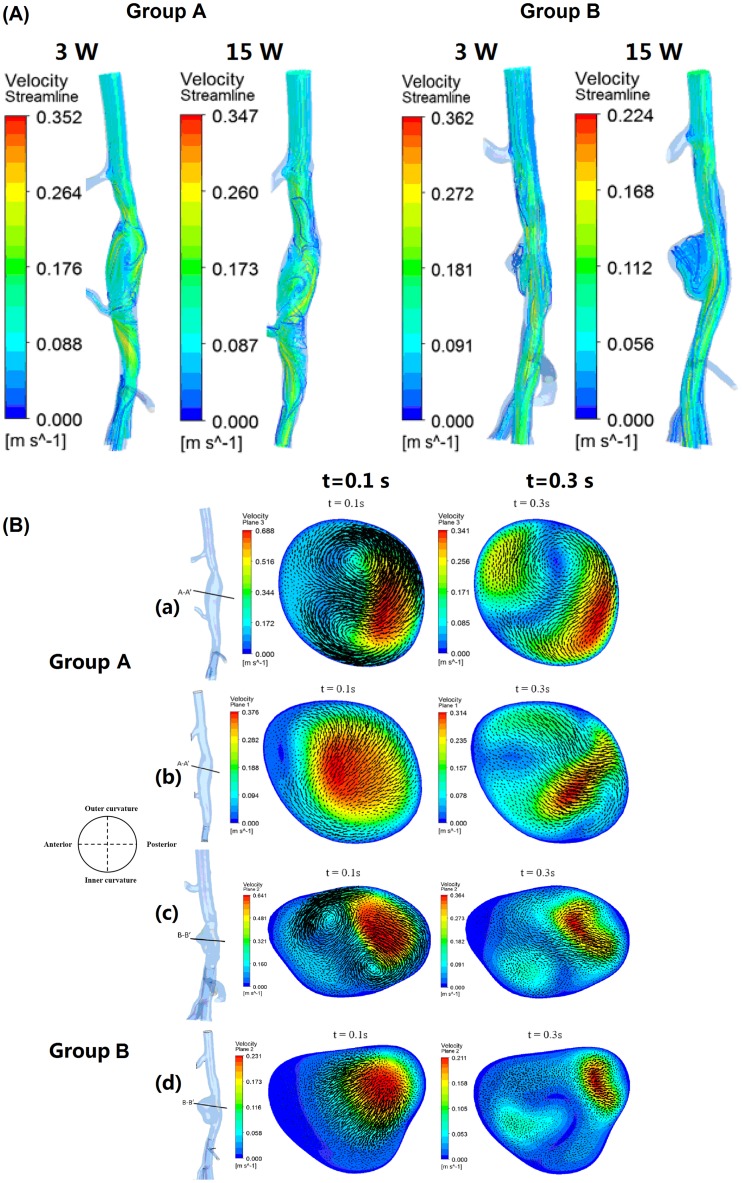
Velocity streamlines at 0.3 s and blood flow patterns at 0.1 s (systolic deceleration) and 0.3 s (diastolic phase) of one cardiac cycle for reconstructed AAA models. (A) In Group A, the velocity magnitude at week 3 was much higher than in week 15. (B) At the section cut A-A’ normal to the centerline, the intensified bulk flow and obvious vortex formation were observed at week 3 instead of week 15. The flow recirculation and vortex formation were randomly distributed throughout the fusiform AAA. However, it shifted towards the anterior region of AAA in Group B. 3 weeks (a, c), 10 weeks (b, d).

**Fig 3:**
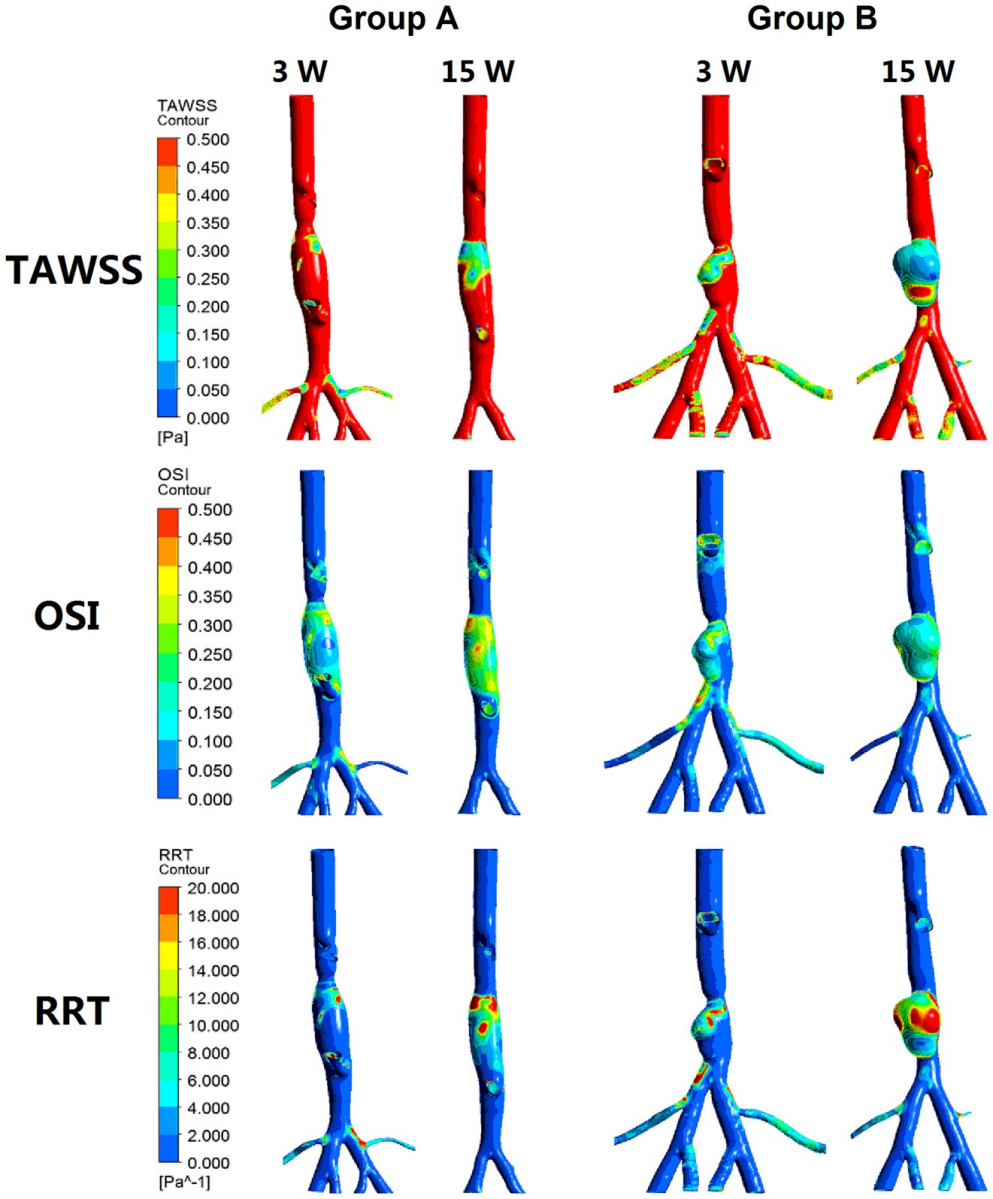
Percentage area of low WSS, high OSI and RRT. Larger Increases in the percentage area of both low WSS (<0.5 Pa) and high RRT (>10 1/Pa) were observed in Group B than Group A. However, the percentage area of high OSI (>0.3) had relatively less increase in Group B than Group A.

In Group A, 2 rabbits died at 8 weeks and 11 weeks, and rabbits were used for hemodynamic analysis at 3 weeks (n = 8) and at 15 weeks (n = 6). Blood flow patterns at 0.1 s (systolic deceleration) and 0.3 s (diastolic phase) of one cardiac cycle for reconstructed rabbit AAA models were shown in Fig 2. In Group A model (Group A), the velocity magnitude at week 3 was much higher than in week 15 due to the aortic coarctation. In addition, at the section cut A-A’ normal to the centerline, the intensified bulk flow and obvious vortex formation were observed at week 3 instead of week 15. The flow recirculation and vortex formation were randomly distributed throughout the fusiform AAA. However, it shifted towards the anterior region of AAA in Group B model. The pressure in Group A model was approximately 47% higher than the one in Group B models. Larger Increases in the percentage area of both low wall shear stress (WSS) (<0.5 Pa) and high relative residence time (RRT) (>10 1/Pa) were observed in Group B than Group A (Fig 3). However, the percentage area of high oscillatory shear index (OSI) (>0.3) had relatively less increase in Group B than Group A.

### Inner diameter follow-up

Compared with Group C, aneurysm diameters in Group A and Group B increased significantly after 1 week (*p* < 0.0001). The aortic diameter in Group A significantly increases up to 3 weeks, then reaches a plateau at 5 weeks and decreased thereafter, probably corresponding to the coarctation relief at 4 weeks. However, rabbit AAA still enlarged gradually in Group B at 15 weeks, which was significantly higher than Group A (*p* < 0.0001). Diameter did not changed significantly in Group C during follow-up (S1 Table).

### Changes in aortic wall thickness

Media thickness of AAA thickened significantly at week 15 (versus week 1, *p* < 0.05) in Group A, which was not significant in Group B. Intimal hyperplasia was obvious in Group A and Group B, intimal thickness increased significantly at weeks 5 and 15 (versus week 1, *p* < 0.01). Additionally, Group A showed a significantly higher intimal hyperplasia compared with Group B (*p* < 0.0001). The intima-media thickness increased significantly in Group A at week 15 compared with Group B and Group C (*p* < 0.01). Group C showed stable intima-media thickness(S2 Table).

### Changes in elastic lamellae and SMCs content

**Fig 4:**
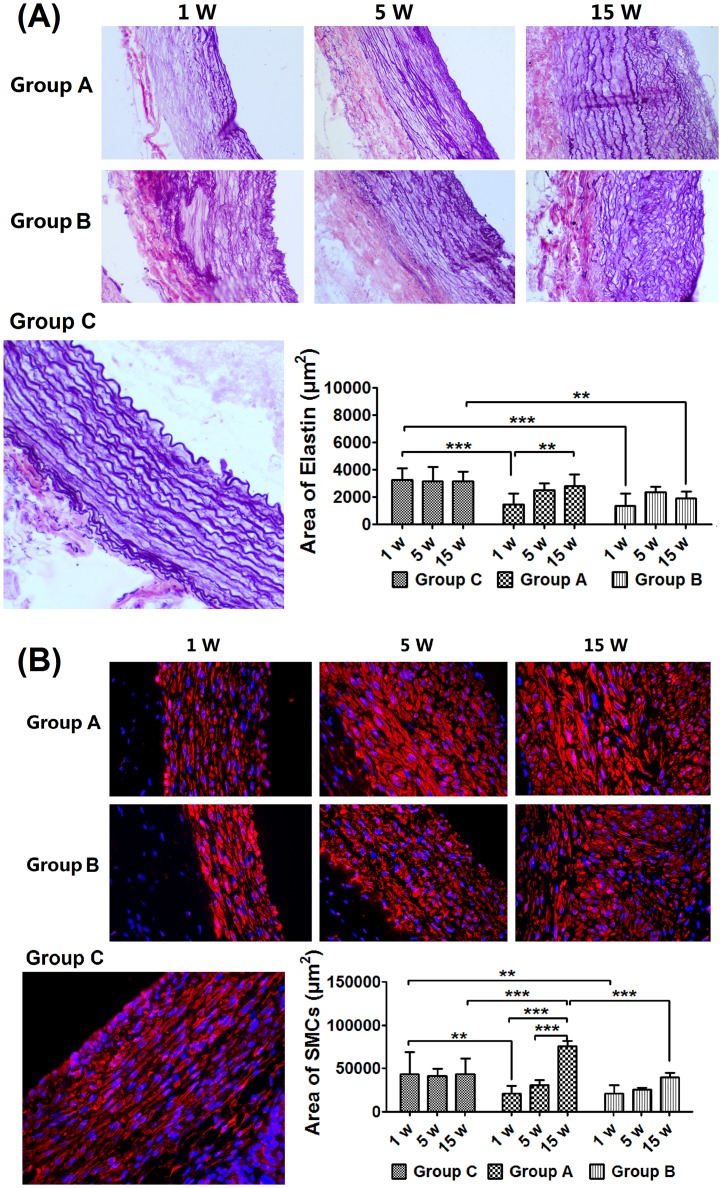
Profiles of elastin content and smooth muscle cells change. (A) Elastin fibers destroyed dramatically at week 1, which increased progressively by week 15 in (B) SMCs destroyed dramatically at week 1, which increased significantly in Group A by week 15 compared with Group B. ** *p* < 0.01, *** *p* < 0.0001.

As shown in Fig 4A, elastic fibers were destroyed markedly in Group A and Group B at week 1 (versus Group C, *p* < 0.0001), which increased to normal level in Group A at week 15. Elastin content remained stable and was lower compared with Group C in Group B at week 15 (*p* < 0.01). The SMCs contents decreased significantly in Group A and Group B at week 1 (versus Group C, *p* < 0.01), and increased thereafter. The SMCs contents increased significantly at week 15 in Group A compared with Group B and Group C (*p* < 0.0001, Fig 4B). The elastin and SMCs content did not changed significantly in Group C(S3 Table).

### MMP2, MMP9 and RAM11 expressions

High-level of MMP9 protein expression was shown at week 1 in Group A and Group B, particularly within inflammatory cells and certain myofibroblasts. MMP9 showed an obvious tendency to decrease at week 15 in Group A and Group B. The RAM11 expression also showed an obvious tendency to decrease at week 15 in Group A and Group B. The MMP2 expression maintained moderate expression in Group A. However, MMP2 expression decreased significantly in Group B at week 15 compared with week 5 and Group A (*p* < 0.01). All expressions did not changed significantly in Group C (S4 Table).

## Discussion

The chemical injury AAA model has been popular used in animal studies. [3–9] Rabbit AAA did not form after 30-min incubation with low concentration elastase solution (0.1–5 units/μl), [3] until aorta destroyed significantly via high concentration elastase. [4] However, elastase induction does not always induce stable AAA and may returned to normal condition soon, [10] namely self-healing phenomenon. [8], To overcome this phenomenon, new kinds of enlarging AAA models were induced in rabbits and rats. [4,13]

In this study, all aneurysms formed at week 3 and enlarged progressively in Group B. The absorbable suture in Group A was dilated 4 weeks later to terminate the aortic coarctation. Interestingly, the aneurysm diameter reaches a plateau at 5 weeks; aneurysms remained stable or even shrunk after coarctation termination in Group A. This indicated that arterial dilation is reversible after removal of coarctation for poststenotic dilatation [17]. The increase rate in Group A was lower than Group B after coarctation relief 4 weeks later, and diameter was significantly lower at week 15 compared with Group B. Elastin fibers and SMCs destroyed dramatically at week 1, and intimal hyperplasia, elastin fibers and SMCs regeneration were found thereafter, especially after coarctation termination in Group A. The different outcomes of AAA models are worthy of investigation, which indicated that hemodynamic change may play an essential role in the initiation and progression AAA in rabbits. [18–20] Specifically, flow disturbance and recirculation pattern was randomly distributed throughout the AAA in Group A, but concentrated in the anterior of AAA sac in Group B. This was associated with the growth rate of the AAA inner diameter [21].

The observed intima thickening with time in both groups could be attributed to the increased percentage area of low WSS and high RRT, the low WSS correlated with the increased intimal thickness [22]. It was observed that a higher blood pressure was correlated with the increased medial thickness [23] and the high OSI was associated with the cell inflammation resulted phenotypic modulation in the initiation and progression of AAA [24]. Interestingly, although the AAA continued to enlarge in Group B, the expression level of MMP2 in Group B was lower than in week 15 in Group A. These complex pathologic and hemodynamic changes may helpful for investigate the pathogenesis of AAA.

Two major shortcomings should be paid attention. There may be an important bias, considering that the material used for the aortic coarctation is different between the groups. To ensure that balloon dilatation completely relieves the coarctation, a absorbable suture was used in Group A, which may induce more inflammation locally and may play a role by itself. Although rabbits in Group B were also ballooned but without correcting the coarctation at the same pressure as the group A at 4 weeks, the injury of balloon procedure may effect on the wall, including increased smooth muscle cell proliferation.

In conclusion, the self-healing of rabbit AAA may attributed to the regeneration of smooth muscle cells. The turbulence flow caused by coarctation is associated with continuous growth of rabbit AAA and prevents the self-healing phenomenon.

## Supporting information

S1 Table Aortic diameter change. (XLSX)

S2 Table Change of aortic wall thickness. (XLSX)

S3 Table Changes in elastic lamellae and vascular SMCs content. (XLSX)

S4 Table Expressions of MMP2, MMP9 and RAM11.1. (XLSX)
